# Foot morphology as a predictor of hallux valgus development in children

**DOI:** 10.1038/s41598-023-36301-2

**Published:** 2023-06-08

**Authors:** Laura Martín-Casado, Alberto Aldana-Caballero, Christian Barquín, Juan José Criado-Álvarez, Begoña Polonio-López, Félix Marcos-Tejedor

**Affiliations:** 1 Department of Nursing, Physiotherapy and Occupational Therapy, Faculty of Health Sciences, University of Castilla-La Mancha, Talavera de la Reina, Toledo Spain; 2 Department of Sport Science, Faculty of Education, Technical University of Ambato, Ambato, Tungurahua Ecuador; 3 Department of Medical Sciences, Faculty of Health Sciences, University of Castilla-La Mancha, Talavera de la Reina, Toledo Spain; 4 Department of Health, Institute of Health Sciences, Talavera de la Reina, Toledo Spain

**Keywords:** Health care, Risk factors

## Abstract

An excess of body weight can produce morphological changes in the feet of children. The aim of this study was to assess the morphological differences of the foot in children based on their body mass index and to determine the risk factors for the development of a hallux valgus in childhood and adolescence. One Thousand Six Hundred Seventy-Eight children (5–17 years) were classified as group with obesity, overweight, and normal weight. Lengths, widths, heights and angles of both feet was measured with a 3D scanner. The risk of developing hallux valgus was calculated. Group with overweight and obesity presented longer feet (p = 0.00), wider metatarsals (p = 0.00) and wider heels (p = 0.00). Arch height was lower (p > 0.01) in the group with obesity, and the hallux angle was greater in the group with normal weight (p < 0.05). The relative risk of a lateral hallux deviation increases with age, foot length and heel width (Exp (B) > 1). Children with overweight and obesity had longer and wider feet. The arch height was higher in children with overweight, and lower in children with obesity. Age, foot length, and heel width could be risk factors for the development of hallux valgus, while metatarsal width and arch height could be protective factors. Monitorization of the development and characterization of the foot in childhood as a clinical tool could help professionals to early identify the patients presenting risk factors and prevent future deformities and other biomechanical conditions in adulthood by implementing protecting measures.

## Introduction

The prevalence of overweight and obesity has increased considerably over the past few years, both in developed countries and in developing countries^1^, which is a worrying condition in children’s health because it interferes with their physiological development^2–4^. As a result of this overweight, children experience musculoskeletal disorders which mainly affect the lower limbs and the feet^5,6^, with frequent alignment disorders, pain in hips, knees and feet, increased risk of fractures, reduced joint flexibility, and gait difficulty^7–10^.

The structure and morphology of the foot can be affected by different factors such as age^11^, shoes^7,12,13^ or body mass index (BMI)^14^. An excess body weight can produce morphological changes and collapse ligaments and supporting structures^2,15^. Previous studies showed bigger feet volumes in overweight and obese children^16^, as well as a decrease in the height of the medial longitudinal arch (MLA), while children with normal weight present narrower feet^15^.

A decrease or absence of MLA is one of the orthopaedic problems most related to excess weight. There is a direct relationship between an increase in body weight and a higher prevalence of flat feet^17^. On the other hand, flat feet are associated with different conditions, such as the development of bunions or hallux valgus deformities in adult population^18,19^. Some authors state that flat feet and excess weight increase the risk of postoperative recurrence of juvenile hallux valgus^20,21^.

Morphological changes in the feet of children with overweight and obesity have been studied before^2,11,14–16^. However, only one study assesses the relationship between excess body weight and a lateral deviation of the first toe in children^2^ and there is not any study addressing the predisposing factors for a valgus injury in the first metatarsophalangeal joint. Therefore, the aim of this study was to assess the morphological differences of the foot in school children based on their BMI and to determine the risk factors for the development of a hallux valgus in childhood and adolescence.

## Methods

### Subjects

This study included 1678 school children (834 boys, 844 girls), aged between 5 and 17, from different Education Units in Ecuador, with a mean weight and height of 37.53 ± 15.46 kg and 1.36 ± 18.4 m, respectively. Parents or tutors signed the informed consent for participation. The study was approved by the Bioethical Committee of the University of San Francisco de Quito (2016-083E), and the Declaration of Helsinki was followed.

Exclusion criteria were: children with clubfoot, metatarsus adductus, visible bunion deformities, os tibiale externum, history of trauma such as sprains and fractures or those who had underwent surgical treatment for these conditions or who had been using plantar insoles as part of a treatment.

### Measurements

The subjects were weighed and measured using a standing scale and height rod model 420KLWA (WelchAllyn, U.S.A.). The BMI was calculated using the Quetelet’s Index (BMI = Weight/Height^2^) and three groups were established, following the classification proposed by Cole et al.^22^, based on BMI, sex and age: normal weight, overweight and obesity. Demographic data of the patients can be seen in Table 1.

**Table 1:** Mean, standard deviation, range, and frequency of demographic characteristics.

	Normal weight (n = 1246)	Overweight (n = 322)	Obesity (n = 113)	*P* value
Age (x̄ ± SD)	10.54 ± 3.60	11.21 ± 3.64	9.34 ± 3.20	0.000
Range [minimum–maximum]	5.00–17.00	5.00–17.00	5.00–17.00	
BMI (x̄ ± SD)	17.79 ± 2.52	22.70 ± 3.20	25.25 ± 4.25	0.000
Range [minimum–maximum]	7.45–24.65	17.45–29.77	19.30–40.01	
Sex (%)				0.214
Girls	38.2	8.8	3.3	
Boys	36.1	10.4	3.3	

x̄ mean, *SD* standard deviation, P value probability value of ANOVA and χ^2^.

**Figure 1:**
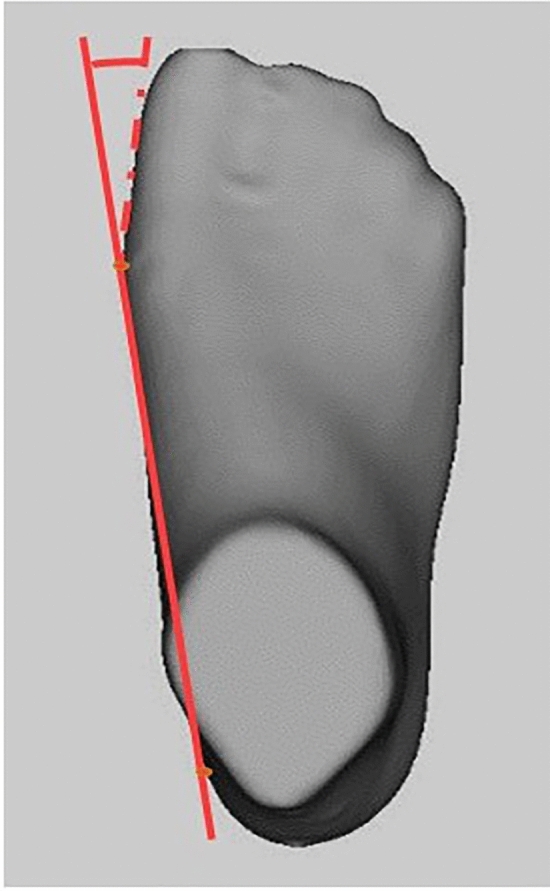
Hallux angle measurement.

The foot anthropometric measurements were:

- Foot length: distance between the heel and the most distal point of the phalanxes.
- Metatarsals width: distance between the most medial point of the first metatarsal head and the most lateral point of the fifth metatarsal head.
- Heel width: distance between the most medial point and the most lateral point of the calcaneus.
- Arch height: distance between the highest point of the plantar arch and the ground.
- Hallux angle: angle between a line from the most medial point of the first toe and the most medial point of the first metatarsal head, and another line from the most medial point of the heel and the most medial point of the first metatarsal head^23^ (Fig. 1). Following the methodology of Kinz et al.^24^, based on this variable, different categories for the position of the first toe were established: varus (− 1°–5°), Straight (0°), Valgus I (1°–5°), Valgus II (6°–10°), Valgus III (11°–15°) and Valgus IV (≥ 16°).

### Protocols

**Figure 2:**
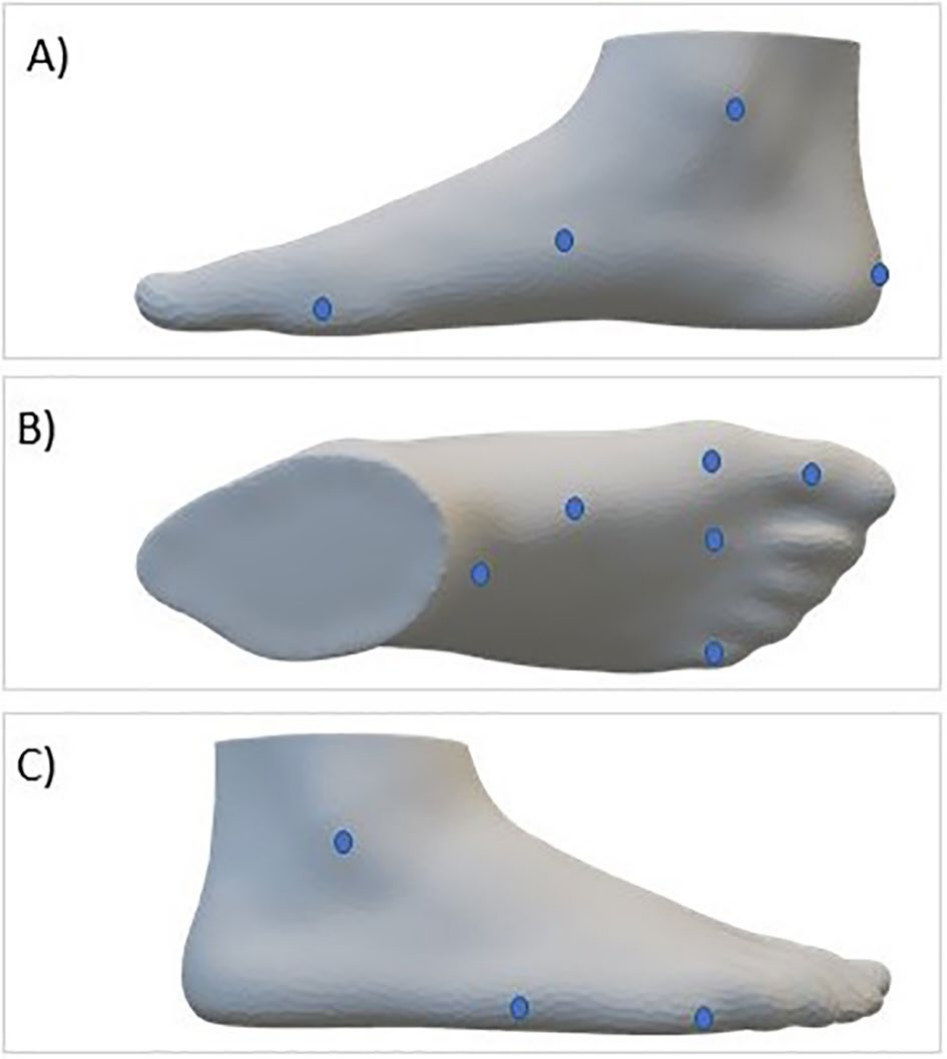
Anatomical landmark placed on the right foot: medial region (**A**), dorsal region (**B**) and lateral region (**C**).

The dimensions of the foot were obtained using the 3D digitizer model IFU-S-01 (INFOOT, Japan). Thirteen anatomical landmarks were placed on the feet of each participant following the manufacturer’s instructions, seated on a chair, with bare feet in neutral position on the floor: metatarsale tibiale, navicular, the most lateral point of medial malleolus, heel born point, first toe joint, head of 2nd metatarsal, highest point of 1st metatarsal head, fifth toe joint, cuneiform, tentative junction point, metatarsale fibulare, the most lateral point of lateral malleolus, tuberosity of the fifth metatarsal (Fig. 2). Later, the left and the right foot were consecutively scanned, in standing position, barefoot, staring straight ahead, and weight evenly distributed on both feet.

### Data analysis

The descriptive analysis was made using descriptive parameters according to the scale of the variable. For the inferential analysis, *Student’s* *t* test was used for dichotomous variables, and nominal and dichotomous variables were compared using *χ*^2^ test. For the analysis of independent variables, *ANOVA* and Bonferroni post-hoc was used to study the relationship between a continuous variable and a nominal variable or to study n independent groups.

In the *multivariate logistic regression analysis,* the association between the variables and the presence or absence of hallux valgus (dependent variable) is calculated using the “Enter” method.

Apart from the absolute measurements, the normalised foot length measurements were also analysed. Confidence Level was set to 5% and statistical analysis was made using SPSS for Windows (Statistical Package Social Sciences version 15.0. Armonk, NY, IBM Corp.).

## Results

In the studied population, normal weight was found in 74.3% of the subjects (n = 1246: 605 boys and 641 girls; BMI = 17.79 ± 2.52 kg/m^2^), overweight in 19.2% (n = 322: 174 boys and 148 girls; BMI = 22.70 ± 3.20 kg/m^2^) and obesity in 6.6% of the subjects (n = 110: 55 boys and 55 girls; BMI = 25.24 ± 4.25 kg/m^2^). Significant differences were obtained for most of the variables between the right and the left foot (p < 0.05), therefore, all the analyses were performed in a total of 3356 feet (1678 right and 1678 left feet).

**Figure 3:**
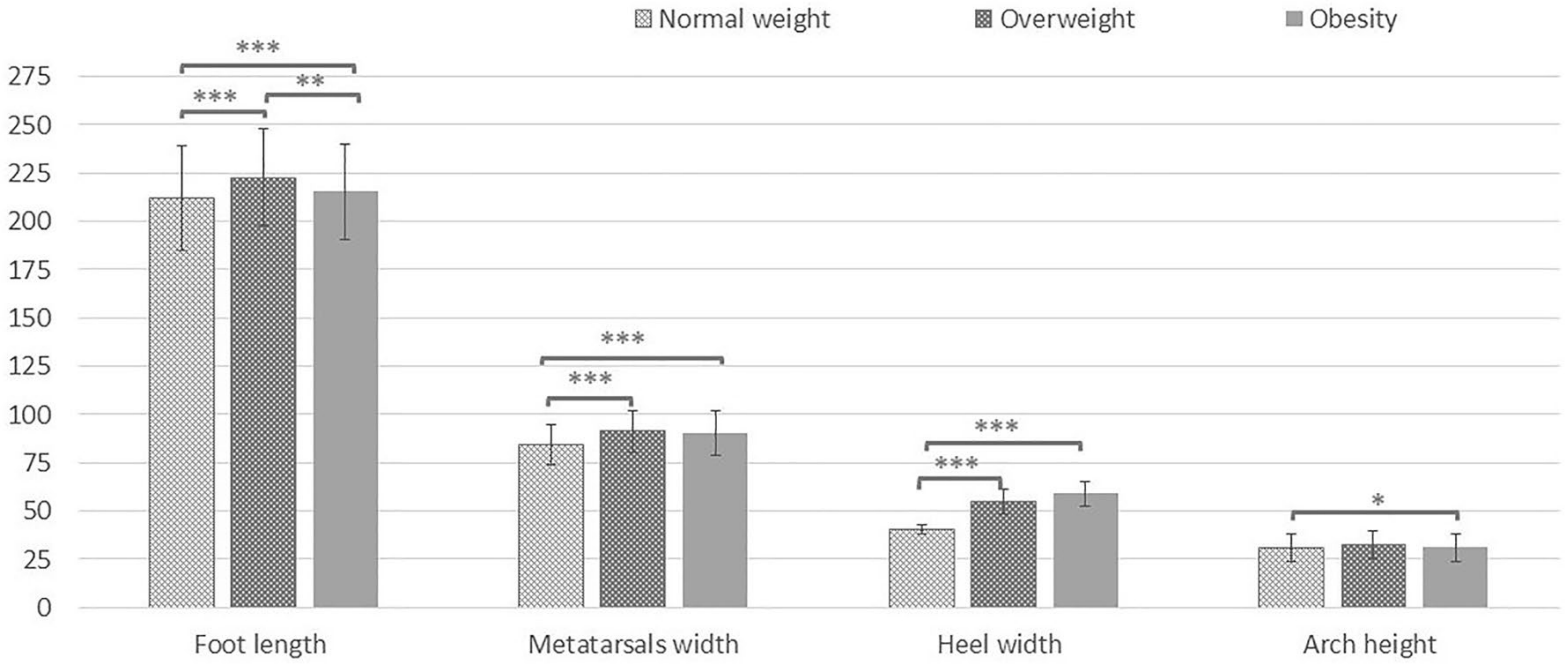
Differences found in variables of foot dimensions (mm) according to BMI. Statistical significance: *p < 0.05, **p < 0.01, ***p < 0.001.

The analysis of the foot dimensions according to BMI showed differences in all the variables examined in the three groups, being higher in boys than in girls (p = 0.00), except the hallux angle, which was higher in normal weight and overweight girls (normal weight: girls = 9.25 ± 4.93° and boys: 8.34 ± 4.87°; overweight: girls = 8.95 ± 4.61°; and boys = 7.70 ± 5.15°; p = 0.00). Longer feet (p = 0.00), wider metatarsals (p = 0.00), and wider heel (p = 0.00) were observed when comparing the overweight and the obesity groups to the normal weight group (Table 2). Arch height was significantly higher in the overweight group compared to the normal weight group (p < 0.01); and the hallux angle was significantly higher (p < 0.05) in the group with normal weight compared to the obesity group (Table 2). When comparing the overweight and the obesity groups, the only difference findings was the length of the foot (p < 0.05) (Fig. 3).

**Table 2:** Mean and SD of the values of foot dimensions according to BMI.

	Normal weight (n = 1246)	Overweight (n = 322)	Obesity (n = 113)	P			
				ANOVA	Bonferroni multiple comparisons		
					Normal weight vs. overweight	Normal weight vs. obesity	Overweight vs. obesity
Foot length (mm)	212.03 ± 27.26	222.65 ± 25.12	215.28 ± 24.77	< 0.001	< 0.001	0.263	0.002
Metatarsals width (mm)	84.48 ± 10.34	91.33 ± 10.88	90.58 ± 11.44	< 0.001	< 0.001	< 0.001	1.000
Heel width (mm)	40.26 ± 2.37	54.89 ± 6.39	59.22 ± 6.34	< 0.001	< 0.001	< 0.001	0.748
Arch height (mm)	31.13 ± 7.34	32.38 ± 7.34	31.00 ± 7.17	0.002	0.001	1.000	0.080
Hallux angle (°)	8.64 ± 5.6	8.27 ± 5.8	7.53 ± 6.33	0.014	0.512	0.018	0.271

P = probability value of ANOVA and Bonferroni post-hoc.

When normalizing the variables of foot dimensions with the foot length, most of the differences found still exists for all the groups.

Based on the established categories of the hallux angle, 5.6% of the subjects presented a varus deviation of the first toe, 3.6% presented a straight toe, and 90.8% a valgus deviation (Table 3).

**Table 3:** Frequency of Hallux angle categories.

Categories of Hallux angles (°)					
Varus	Straight	Valgus			
		1	II	III	IV
1–5°	0°	1–5°	6–10°	11–15°	≥ 16°
5.6%	3.6%	20.5%	37.1%	24.8%	8.4%

When the BMI is analysed based on the degree of valgus deviation of the first toe, there are significant differences between straight position and IV valgus (p = 0.032) with a mean difference of 1.27 between the groups (Straight = 19.94 kg/m^2^; valgus IV = 18.67 kg/m^2^). Valgus deviation was more frequent in girls than in boys (*χ*^2^ = 13.572; df = 1, p = 0.001), and significant differences were found in arch height between the hallux valgus categories (p = 0.00).

**Figure 4:**
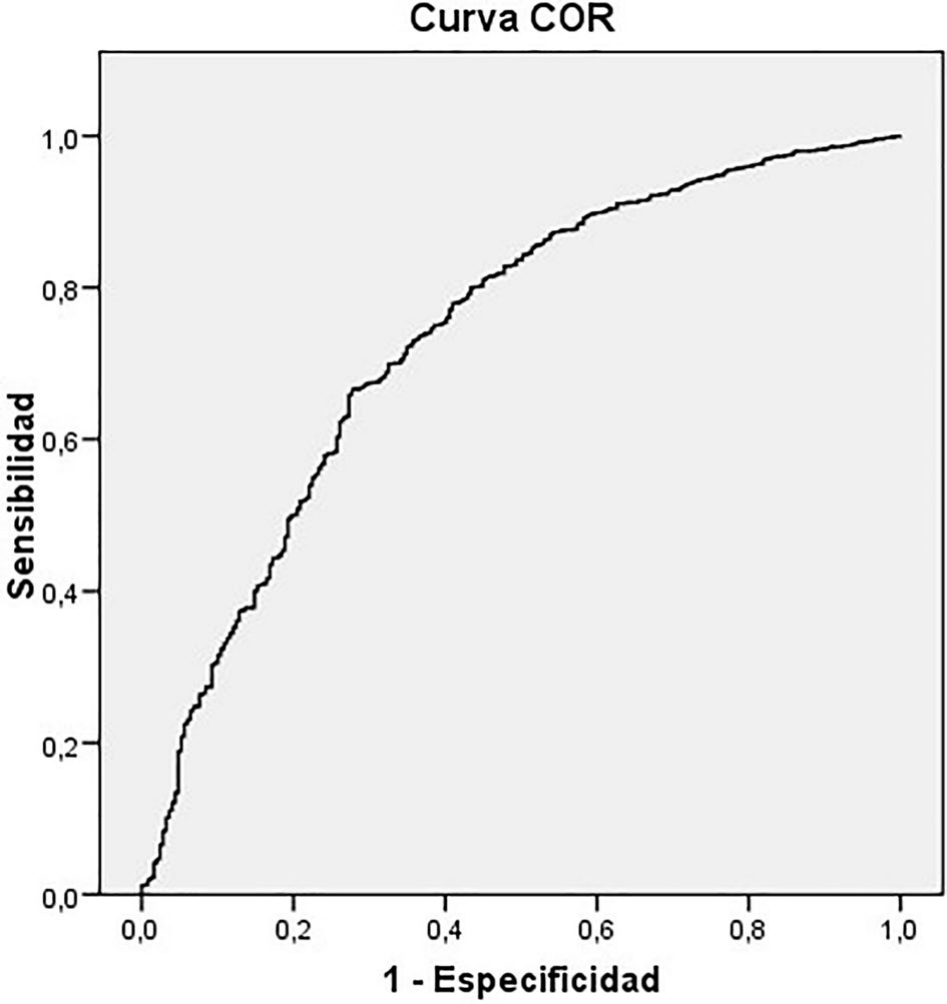
The ROC curve multivariate logistic regression analysis.

The multivariate logistic regression analysis, with the presence of a deviation in valgus of the first metatarsophalangeal joint (Categories I, II, III and IV of hallux valgus angles) as the dependent variable, showed statistically significant results (p < 0.05). The model correctly classifies 90.6% of the cases, with age, foot length, and heel width being risk factors for the valgus deviation of the first toe (Exp (B) > 1), while sex, metatarsal width and arch height are protective factors (Exp (B) < 1) (Table 4). The area under the ROC curve is 0.736 (CI95%: 0.702–0.771). The ROC curve is shown in the Fig. 4.

**Table 4:** Multivariate logistic regression analysis results.

	B	Sig	Exp (B)	95% CI for EXP(B)	
				Lower	Upper
Age	0.114	0.004	1.120	1.036	1.212
Sex	− 0.408	0.006	0.665	0.930	0.964
Foot length	0.032	0.000	1.032	1.016	1.048
Metatarsal width	− 0.155	0.000	0.857	0.831	0.883
Heel width	0.093	0.001	1.097	1.039	1.159
Arch height	− 0.052	0.000	0.949	0.932	0.966
Constant	4.346	0.000	77.139		

B beta coefficient, Exp (B) beta exponential.

*95*% *CI* 95% confidence interval.

## Discussion

Although the prevalence of overweight and obesity in Ecuadorian children is lower compared to other studies performed in Mexican population^17^ or European population^14,16^, the numbers keep growing in the last couple of years, and the effects of obesity on health cannot be ignored, as these effects are of paramount importance during the development in childhood and adolescence for the consequences it might have in adulthood^25^.

The results in our study indicate that overweight and obese children present longer, and wider feet compared to children with normal weight, who present shorter and narrower feet (Table 2). These results are in line with other studies, which observed longer and wider feet in children with excess weight^14–16^. Most of the differences disappear between the overweight and the obesity groups (Table 2). With an increase in body weight, differences on the dimensions of the foot arise. However, the lack of differences between these two groups could suggest the existence of a weight threshold from which the foot in children start showing changes in its dimensions, with structural transformations, that modify the body weight distribution and the mechanical load, affecting the normal function of the foot. These changes alongside with an excess in body weight can have an impact in the physiological development. Therefore, it is important to monitor the development and characterize children’s feet in the initial stages of overweight as a clinical tool, that will allow for an early implementation of physical or orthotic treatments to prevent future deformities and other problems on the foot related with obesity in adulthood.

The highest values for the plantar arch were found in overweight children and the lowest values in children with obesity (Table 2). Although most studies correlate an excess weight with a decrease of the MLA^11,26–30^, others do not find differences^14,31^. The height of the arch can be modified not only by the BMI but also by age, which determines the physiological development in children^26^. In our study, the mean age of obese children was 9.3 ± 3.6 years, while in overweight children was 11.2 ± 3.6 years (p < 0.05). This difference in age between these groups could explain the fact that overweight children, who are older, presented a higher arch height, because the natural growth and maturity of the bone and supporting structures is independent of the body weight. With the methods used, it is not possible to determine whether the differences in the arch height are due to the bone or adipose structures of the foot.

When normalizing the variables to the foot length, the differences between the three groups are maintained, which is in line with most of the studies that analyze the morphology of the standing foot^11,15,16^. However, some authors do not find differences between the groups when normalizing the foot measurements to the foot length^14^.

According to the results of our study, the main risk factors for the development of a hallux valgus are age (OR = 1.12, 95% CI 1.03–1.21, p = 0.004), followed by heel width (OR = 1.09, 95% CI 1.03–1.15, p = 0.001) and foot length (OR = 1.03, 95% CI 1.01–1.04, p = 0.00). In older children, as the foot gets longer and wider, it exists a higher risk of a hallux valgus injury. The bearing loads that the foot supports are distributed following the bearing surface, our results indicate that overweight and obese children have longer and wider feet (Table 2), which supposes an increment in bearing forces, especially over the rearfoot, and displacing the axis of the STJ medially in motion, therefore, impeding an optimal movement of the metatarsophalangeal joint during the toe-off phase of the gait. In addition, the hallux angle was 10.32% higher in girls than in boys (p = 0.00), and sex was found to be a predicting factor for boys (OR = 0.66, 95% CI 0.49–0.89, p = 0.006). These results are in line with the results obtained by other studies in which higher angles on the first toe were found in girls between 16 and 17 years old, fact that is mainly attributed to the use of enclosed shoes in colder areas^12^. Other studies observed a higher prevalence of hallux valgus in adult women with square foot, who used narrow toe box^13^; there is a direct relationship between a poor shoe adjustment and a hallux valgus deviation^7,24^. Environmental conditions and preferred shoe type are factors that can affect the position of the first toe, but these were not considered in this work. The growth in length and width of the foot of girls according to age and the use of a shoe type that encloses the forefoot could increase the risk of deformity in the first metatarsophalangeal joint. In fact, the metatarsal width is a protective factor against a hallux valgus deviation (OR = 0.85, 95% CI 0.83–0.88, p = 0), therefore, wider feet are at less risk of developing this. No direct relationship between the BMI and the different hallux valgus categories has been found in our sample. A 10.9% of the children with obesity presented straight hallux angle and only 4.1% presented IV valgus (Table 5). On the other hand, children with straight hallux angle also had higher plantar arches compared to children with IV valgus (straight: 33.24 ± 7.74 cm; valgus IV: 29.71 ± 6.84 cm).

**Table 5:** Frequency (in %) of different categories of hallux angles in children in relation to their BMI.

Categories of hallux angles	Normal weight (n = 1246)	Overweight (n = 322)	Obesity (n = 113)
Varus	72.8	18.5	8.7
%^a^	4.1	1.0	0.5
Straight	68.1	21.0	10.9
%^b^	2.5	0.8	0.4
Valgus I	73.8	19.4	6.8
%^c^	15.1	4.0	1.4
Valgus II	72.6	20.7	6.7
%^d^	26.7	7.7	2.5
Valgus III	77.2	17.2	5.6
%^e^	19.2	4.3	1.4
Valgus IV	78.7	16.2	5.1
%^f^	6.6	1.4	0.4

^a^Relative frequencies in percentages of all participants included in the study with varus hallux angle.

^b^Relative frequencies in percentages of all participants included in the study with straight hallux angle.

^c^Relative frequencies in percentages of all participants included in the study with Valgus I hallux angle.

^d^Relative frequencies in percentages of all participants included in the study with Valgus II hallux angle.

^e^Relative frequencies in percentages of all participants included in the study with Valgus III hallux angle.

^f^Relative frequencies in percentages of all participants included in the study with Valgus IV hallux angle.

Similar results were obtained in another study^2^, which did not find differences between the BMI and the hallux angle in children aged 11–13. However, there is no agreement on the relationship between flat feet and hallux valgus. Some authors correlate flat feet with juvenile hallux valgus, while others do not see any relationship between flat feet and the degree of hallux valgus deformity^18,21,32^. This study suggests that the arch height is a protective factor against a hallux valgus deviation (OR = 0.94, 95% CI 0.93–0.96, p = 0) (Table 4). The fact that medial longitudinal arches are higher in girls and boys with a straight hallux could indicate that MLA’s during childhood and adolescence, regardless of the body weight, could protect against a future hallux valgus.

With these results, considering the limitations of the study, it is suggested that in future research, intrinsic factors such as genetics, the type of feet based on the length of the toes, soft tissue or bone structure should be taken into consideration, and assess if the differences found in the dimensions of the foot in relation to the BMI found in this study are only related to the adipose structure of the foot. In addition, in the present study, it could not be measured the changes on the foot between the unloaded and the loaded position, that could throw important information about if the body weight is a direct factor on conditions of the foot during childhood and adolescence. Lastly, extrinsic factors such as shoes and other habits that could affect the position of the first toe, should be also considered.

## Conclusions

Excess body weight affects the dimensions of the foot during childhood and adolescence. Overweight and obese children presented longer, and wider feet compared to normal weight children. The highest values in plantar arches were observed in overweight children and the lowest values in obese children. Age, foot length, and heel width could be risk factors for the development of hallux valgus during childhood and adolescence, while metatarsal width and arch height could be protective factors. Monitorization of the development and characterization of the foot in childhood as a clinical tool could help professionals to early identify the patients presenting risk factors and prevent future deformities and other biomechanical conditions in adulthood by implementing protecting measures.

## Data Availability

All data generated or analysed during this study are included in this published article.
